# Stress induced a shift from dorsal hippocampus to prefrontal cortex dependent memory retrieval: role of regional corticosterone

**DOI:** 10.3389/fnbeh.2014.00166

**Published:** 2014-05-15

**Authors:** Gaelle Dominguez, Pierre Faucher, Nadia Henkous, Ali Krazem, Christophe Piérard, Daniel Béracochéa

**Affiliations:** ^1^INSERM U-930, Université François Rabelais, Parc GrandmontTours, France; ^2^Institut de Neurosciences Cognitives et Intégratives d'Aquitaine, CNRS UMR 5287, Nouvelle Université de BordeauxTalence, France; ^3^IRBA - Unité Neuropsychopharmacologie - Brétigny sur OrgeFrance

**Keywords:** stress, glucocorticoids, microdialysis, hippocampus, prefrontal cortex

## Abstract

Most of the deleterious effects of stress on memory retrieval are due to a dysfunction of the hippocampo-prefrontal cortex interplay. The role of the stress-induced regional corticosterone increase in such dysfunction remains however unclear, since there is no published study as yet dedicated to measuring corticosterone concentrations simultaneously in both the prefrontal cortex (mPFC) and the hippocampus (dHPC) in relation with memory impairments. To that aim, we first showed in Experiment 1 that an acute stress (3 electric footschocks; 0.9 mA each) delivered before memory testing reversed the memory retrieval pattern (MRP) in a serial discrimination task in which mice learned two successive discriminations. More precisely, whereas non-stressed animals remembered accurately the first learned discrimination and not the second one, stressed mice remembered more accurately the second discrimination but not the first one. We demonstrated that local inactivation of dHPC or mPFC with the anesthetic lidocaine recruited the dHPC activity in non-stress conditions whereas the stress-induced MRP inversion recruited the mPFC activity. In a second experiment, we showed that acute stress induced a very similar time-course evolution of corticosterone rises within both the mPFC and dHPC. In a 3rd experiment, we found however that *in situ* injections of corticosterone either within the mPFC or the dHPC before memory testing favored the emergence of the mPFC-dependent MRP but blocked the emergence of the dHPC-dependent one. Overall, our study evidences that the simultaneous increase of corticosterone after stress in both areas induces a shift from dHPC (non-stress condition) to mPFC-dependent MRP and that corticosterone is critically involved in mediating the deleterious effects of stress on cognitive functions involving the mPFC-HPC interplay.

## Introduction

Pathological states of memory encountered in stress-related disorders are mainly linked to dysfunction of the mPFC-HPC interplay. Thus, glucocorticoid (GR) and mineralocorticoid receptors (MR), which exhibit a different affinity for corticosterone, are heavily expressed in the hippocampus, the amygdala and medial prefrontal cortex (mPFC) (Reul and de Kloet, 1985; de Kloet et al., 1986; Van Eekelen et al., 1988). Given the localization of GRs receptors, memory impairments induced by exposure to a stressor or glucocorticoids (GCs) are mainly correlated to altered plasticity into the hippocampus, the amygdala and the prefrontal cortex (Maroun and Richter-Levin, 2003; Jay et al., 2004; Vouimba et al., 2004; Sandi et al., 2005).

From a cognitive point of view, cortisol-induced deficits in declarative memory retrieval are associated with a decrease in hippocampal activity in humans, (de Quervain et al., 2003) alike corticosterone-induced deficits in rodents (de Quervain et al., 1998; Roozendaal, 2002; Roozendaal et al., 2003, 2004a,b). Endogenous GCs have also been found to bear an essential role in maintaining prefrontal cortical cognitive functions, mainly via an interaction with dopaminergic and glutamatergic receptors (Mizoguchi et al., 2003, 2004; Yuen et al., 2009). The impact of GCs on cognitive functions is however not uniform. GCs effects on memory processes tend *either* to occur gradually over time via transcriptional regulation initiated by intracellular receptor activation (McEwen and Sapolsky, 1995; McGaugh and Roozendaal, 2002; Joels et al., 2006) *or* may develop rapidly along a non-genomic pathway through membrane receptor activation (Borski, 2000; Falkenstein et al., 2000; Caudal et al., 2010; Chauveau et al., 2010; Conboy and Sandi, 2010; Chaouloff and Groc, 2011; Dorey et al., 2011). Recent findings have underscored striking shifts in the levels of both MRs and GRs that varied by brain regions (Segal et al., 2010) but also time after stress. More specifically, we recently reported a triple dissociation as regards the time-course involvement of the hippocampal regions, corticosterone rises and glucocorticoid receptor types in relation with memory retrieval impairments after acute stress (Dorey et al., 2012).

It is noteworthy that we previously provided unequivocal evidence to the effect that the dHPC and mPFC simultaneously interact at the time of retrieval in a contextual serial discrimination task (CSD). Thus, we evidenced that an acute stress delivered before memory testing reversed the memory retrieval pattern (MRP) in a serial discrimination task in which mice learned two successive discriminations. More pointedly, whereas non-stressed animals remembered accurately the first learned discrimination and not the second one within a series, stressed mice remembered more accurately the second discrimination but not the first one. Moreover, the MRP under non-stress conditions was critically dependent on the dHPC but not on mPFC, whereas the opposite was observed under stress conditions (Chauveau et al., 2008, 2009, 2010; Tronche et al., 2010).

The role of corticosterone in the stress-induced MRP inversion is however not known. Indeed, a previous study from our team has shown that an i.p. metyrapone injection (an inhibitor of the synthesis of corticosterone) before stress delivery totally blocked the stress-induced inversion of the MRP (Chauveau et al., 2010). This result evidenced that corticosterone is crucial to induce the memory retrieval dysfunction resulting from stress delivery in the CSD task. However, we did not investigate as yet the role of the stress-induced regional corticosterone increases in the emergence of the mPFC-dependent pattern after stress and the concomitant blockade of the dHPC-dependent one.

Hence, insofar as our earlier results were collected from lesioned animals, we elected therefore to further probe in an additional experiment the effect of local inactivation of either the mPFC or dHPC by *in situ* infusion of the anesthetic lidocaine (a sodium chanel blocker) on MRP, with a view to eliciting supplementary evidence as regards their distinctive involvement on MRPs under stress and non-stress conditions, respectively. In Experiment 2, using double intracerebral microdialysis, we measured the time-course evolutions of corticosterone rises after stress delivery *simultaneously* into the mPFC and dHPC in the same animal. Given the data obtained, we performed in Experiment 3, direct injections of corticosterone into either the mPFC or dHPC before memory testing, to ascertain the impact of the regional increase of corticosterone concentrations in each brain area on MRP.

## Materials and methods

### Animals

Animals were 6 month-old naive male mice of the C57 Bl6/J inbred strain obtained from Charles River (L'Arbresle, France). They were randomly assigned to the various behavioral experiments. Animal weight ranged between 28 and 32 g. They were housed individually with free access to food and water on a 12 h light-dark cycle in a temperature controlled and ventilated room. All procedures were conducted during the light phase of the cycle between 08.00 a.m. and 12.00 p.m. All subjects were maintained at 85–90% of their *ad libitum* body weight throughout the behavioral study.

All procedures complied with the European Communities Council Directive 2010/63/EU for animal experiments.

### Behavioral test

**Figure 1:**
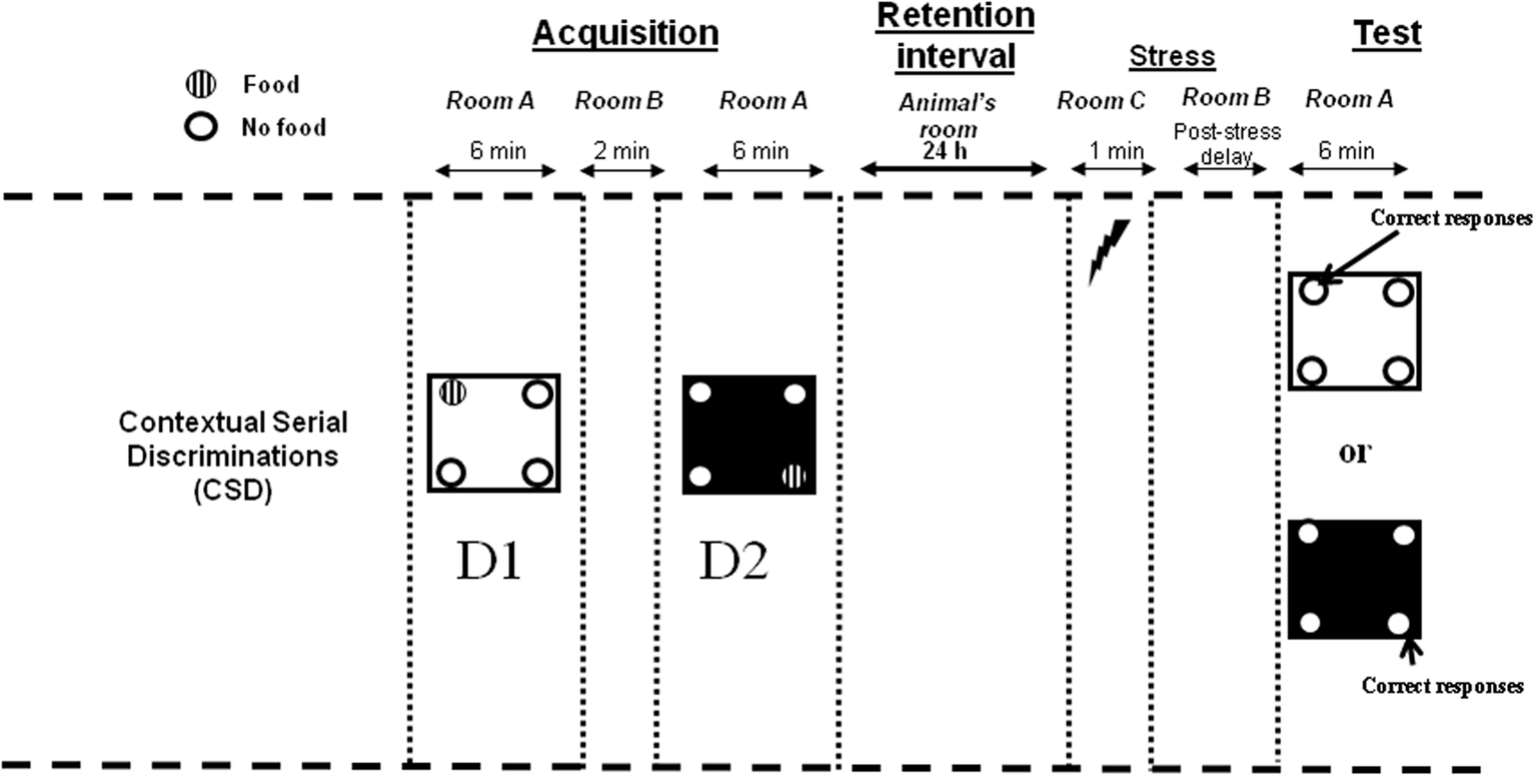
**Contextual Serial Discrimination: at the acquisition phase, mice performed two consecutive spatial discriminations varying by the color and texture of the floor, i.e., D1: Discrimination 1 and D2: Discrimination 2**. For each discrimination, only one hole out of the 4 holes of the apparatus was baited (hashed circles). A 24-h delay was interpolated between the acquisition and test phases, during which mice were returned in the animal room. Fifteen minute (post-stress delay) prior to behavioral testing, mice received an electric footshock in a chamber placed in a room (room C) different from the one in which the behavioral experiments was conducted (room A). Subsequently, mice were submitted to the test phase in which they were replaced either on the floor of the first or the second discrimination without any food pellet in the apparatus. Compound injections occurred 30 min (Experiment 1) or 15 min (Experiment 3) before behavioral testing.

The experimental design is described in Figure 1.

### Apparatus

#### The hole-board

All tests were performed in a four-hole board apparatus (45 × 45 × 30 cm high) enclosed by gray Plexiglas. The four-hole board apparatus was placed on the floor of the room (3.0 × 3.0 × 2.40 m high). The floor of the board was interchangeable (white and smooth; black and rough). On the floor, 4 holes opening on a food cup (3 cm diameter × 2.5 cm in depth) were located 6 cm away from the sidewalls. The apparatus was placed in a room exposed to a 60 dB background noise and a light centered over the apparatus provided 20 lux intensity at the position of the apparatus. The environmental spatial cues were made of colored and striped paper sheets stuck on the walls of the room, and positioned at 1.00 m above the floor. These allocentric spatial cues remained at the same place throughout the acquisition and memory retrieval testing phases. The apparatus was cleaned with 70% ethanol and then with water before each mouse behavioral testing. Photocells placed in each hole were used to evaluate the number of head-dips in the 4 holes.

#### The stress chamber

Stress was delivered in a stress chamber (20 × 15 × 15 cm) which was enclosed with Plexiglas walls, one transparent and the three others painted brown. The floor of the conditioning chamber consisted of 35 stainless steel rods (3 mm diameter), spaced 5 mm apart and wired to a shock generator for the delivery of the three successive foot-shocks (0.9 mA; 1 s). Mice were placed in the conditioning chamber for 1 min and received three successive electric footshocks after 10, 30, and 50 s. The stress chamber was placed in a different room (room C) from the one used for the learning task (room A; see Figure 1) and was cleaned with 95% ethanol then with water between each mouse.

***Contextual and Serial Discrimination task (CSD) protocol***. Behavioral memory testing was conducted in room A (Figure 1). For each discrimination, mice were first placed at the center of the board in a PVC tube for 15 s. Subsequently, the PVC tube was removed and mice could freely explore the four-hole board, with one hole being baited out of the 4 holes in the board. Mice learned two successive spatial discriminations (first discrimination, D1 and second discrimination, D2) for 6 min each. The two serial discriminations differed by the floor color and texture (white and smooth vs. black and rough), and were separated each by a 2 min time interval during which the mouse was placed in its home cage in room B. Further, the sequencing of the two different floors in the series (1st vs. 2nd discrimination) was systematically alternated from one mouse to another within each group. For discrimination 1, 10 20 mg pellets were available only in one of the four holes in the board. The baited hole for discrimination 1 was chosen at random. For discrimination 2, 10 20 mg pellets were systematically located in the diagonally opposite hole. Subjects which did not eat at least 8 pellets at D1 and D2 within the 6 min period were discarded from analysis.

#### Behavioral analysis

In the retrieval phase, for each discrimination, the percentage of “correct responses” was considered to measure memory (number of explorations into the previously baited hole of the same internal (floor) context/total number of explorations X 100.; see Figure 1).

## Experiment 1

The number of animals per group for memory testing in discriminations 1 and 2 was as follows: Lidocaine + Stress; *N* = 10; Lidocaine non-stress; *N* = 8; Vehicle + stress, *N* = 8; Vehicle non-stress, *N* = 8.

### Surgery

Subjects were anesthetized with a mixture of ketamine (100 mg/kg; Panpharma) and xylazine (10 mg/kg; Sigma) injected i.p. and then placed in a stereotaxic apparatus (Kopf). Xylocaine (5%; AstraZeneca) was applied locally before opening the scalp and trepanation. Animals were bilaterally implanted in the prelimbic cortex (termed in this study as “mPFC”) with stainless steel guide cannulae (length: 8 mm; outer diameter 0.46 mm; inner diameter 0.255 mm; Le Guellec tubular components, France), according to the following stereotaxic coordinates (Paxinos and Franklin, 2001): anteroposterior relative to bregma, AP: +1.78 mm; lateral to the sagittal line, L: ±0.3 mm; ventral from the skull surface, V: −1.25 mm. A similar procedure was used for dHPC implantations at the following coordinates: AP = −2.0 mm; *L* = ±1.4 mm; *V* = −0.9 mm. The incisor bar was leveled with the interaural line. Sterile stylets were inserted in the cannulae to maintain patency. Mice were allowed to recover from surgery for 2 weeks before behavioral testing. Mice of the sham-operated groups underwent the same surgical procedures, and received the same amount of the vehicle solution. Guide-cannulae were fixed in place with dental cement and three micro screws attached to the skull.

### Injection procedure

Lidocaine (0.06 nmol/0.3 mL; Sigma) was dissolved in PBS (pH 7.4) (Vandesquille et al., 2013). All animals were given the vehicle solution (artificial cerebrospinal fluid, aCSF; Phymep) 30 min before the acquisition phase.

During the test phase, all mice were gently restrained while the stylets were removed and replaced with the injection needles that extended 2.25 mm beyond the skull surface. The vehicle or lidocaine solutions were bilaterally infused 30 min before behavioral testing using a syringe pump (Braun Perfusor VI; Roucaire) set at a flow rate of 0.1 μL/min, using 1 μL Hamilton syringes. Needles were kept in place for 5 min after completion of the infusion to avoid back up into guide cannulae, and to enhance local spreading of the solutions within the tissue.

## Experiment 2: plasma and regional corticosterone concentrations using double-microdialysis

The experiments were conducted between 8:00 and 11:00 AM. Plasma corticosterone concentrations were measured on independent groups of mice at either 15, 30, 60, 90, 105, or 120 min post-stress delay intervals (7 mice per group). Control animals (*N* = 7) were submitted to the same experimental conditions but did not receive electric footshocks. The time course evolution of corticosterone concentrations (microdialysis experiment) was measured on 7 animals.

### Surgery

Subjects were anesthetized with a mixture of ketamine (100 mg/kg; Panpharma) and xylazine (10 mg/kg; Sigma) injected i.p. and then placed in a stereotaxic apparatus (Kopf). Xylocaine (5%; AstraZeneca) was applied locally before opening the scalp and trepanation.

Two microdialysis guide-cannulae (CMA/7 Microdialysis probe, CMA Microdialysis, Sweden) were implanted at the following coordinates from the bregma (Paxinos and Franklin, 2001): mPFC: AP: +1.8 mm; L: ±0.3 mm; V: −1.25 mm; for dHPC: AP = −2.0 mm; *L* = ±1.3 mm; *V* = −1.0 mm. The laterality of implantation in dHPC and mPFC regions was randomized since half of the animal was implanted in the right side for dHPC and left side for mPFC whereas the other half was implanted in the opposite way.

Guide-cannulae were fixed with dental cement and three micro screws attached to the skull. All operated mice were allowed to recover for 15 days in the animal room. The day before the experiment, the microdialysis probes were introduced through the guide-cannulae and lowered 1 mm below so that the microdialysis membrane was located into dHPC or mPFC. This protocol allows the mouse to become familiar with the microdialysis bowl and whole microdialysate set up. During the night, mice were continuously perfused with sterile filtered Dulbecco's solution (mock CSF) at a rate of 0.1 μl/min.

#### Microdialysis

The experiment was carried out between 8:00 and 11:00 AM. Microdialysis was performed in freely moving animals to determine corticosterone levels in dHPC and mPFC after acute stress. All animals were food deprived for the behavioral and immunohistological studies at the time of measurements. The acute stress was applied in the microdialysis bowl and involved the same 3 successive unavoidable electric footshocks as exerted in the framework of the behavioral experiments. On the day of microdialysis measurements, probes (CMA/7, membrane length 1 mm; CMA Microdialysis, Sweden) were continuously perfused with sterile filtered Dulbecco's solution (mock CSF) at a rate of 1.0 μl/min. After this equilibration phase, baseline dialysates (15 min samples) were collected with a flow rate of 1.0 μl/min during 1 h. Then, dialysates were collected during 2 additional hours after stress (flow rate: 1 μl/min; sampling delay: 15 min). Samples were stored at −80°C before analysis. Free corticosterone levels measured in the dialysates were expressed in relative concentrations as the percentage of the 3 averaged baseline values.

#### Intra-hippocampal and mPFC corticosterone assays

An Enzyme Immunoassay commercial kit (Correlate-EIA™, Assay Designs, Ann Arbor, USA) was used to measure dHPC and mPFC corticosterone concentrations in the microdialysates. The sensitivity of the assay was 18.6 pg/ml. Therefore, baseline sample concentration was more than 10-fold above the sensitivity threshold.

#### Plasma corticosterone measurements

All animals were food deprived similarly to the behavioral studies. Mice were placed in a footshocks delivery system located in the microdialysis bowl, for at least 1 h before being administered with the same electric footshocks used in the behavioral and microdyalisis experiments. After stress, they remained in the microdialysis cage and were decapitated to collect trunk blood after either 15, 30, 60, 90, 105, or 120 min post-stress delays. Control animals were submitted to the same experimental conditions but did not receive electric footshocks. They were killed 1 h after being placed in the microdialysis bowl. Thus, these animals constitute a “time 0” control group. After centrifugation at 3000 r.p.m. for 10 min, the supernatant was stored at −80°C until ELISA assay (Correlate-EIA, Assay Designs, Ann Arbor, USA).

## Experiment 3: effects of intracerebral corticosterone injections into the mPFC or dHPC on memory patterns

This experiment aimed at determining whether intra-hippocampus or intra-PFC corticosterone injections produced a modification of the MRP, similar to that induced by the acute stressor in the CSD task. For both discriminations 1 and 2, memory performance of corticosterone-injected mice into the dHPC (*N* = 9) was compared to groups receiving the vehicle solution (*N* = 9). Memory performance for discrimination 1 of corticosterone-injected mice into the mPFC was compared to a vehicle group (*N* = 10 and 9, respectively); similarly, for discrimination 2, corticosterone-injected group was compared to a vehicle—injected group (*N* = 9 in both cases).

As in Experiment 2, the same anesthetic and surgical procedures were used for surgery. Animals were implanted bilaterally with stainless steel guide cannulae (length: 8 mm; outer diameter 0.46 mm; inner diameter 0.255 mm; Le Guellec tubular components, France), according to the following stereotaxic coordinates (Paxinos and Franklin, 2001): Prelimbic cortex. anteroposterior relative to bregma, AP: +1.78 mm; lateral to the sagittal line, L: ±0.3 mm; ventral from the skull surface; V: −1.25 mm. For dHPC: AP = −2.0 mm; *L* = ±1.4 mm; *V* = −0.9 mm. The incisor bar was leveled with the interaural line. Sterile stylets were inserted in the cannulae to maintain patency. Mice were allowed to recover from surgery for 2 weeks before behavioral testing. Mice of the sham-operated groups underwent the same surgical procedures, and received the same amount of the vehicle solution. Guide-cannulae were fixed in place with dental cement and three micro screws attached to the skull.

The corticosterone dose was chosen according to previous studies from our team (Dorey et al., 2012; Minni et al., 2012; Moisan et al., 2014). Corticosterone (Sigma, France) was diluted in an artificial cerebrospinal fluid at the concentration of 1 mg/ml, and bilaterally injected (0.5 μl per side into the dHPC and 0.4 μl per side into the mPFC). The vehicle and corticosterone solutions were bilaterally infused using a syringe pump (Braun Perfusor VI; Roucaire) set at a flow rate of 0.1 μL/min, using 1 μL Hamilton syringes. Needles were kept in place for 5 min after completion of the infusion to avoid back up into guide cannulae.

In the acquisition phase, all animals were given the vehicle solution 15 min before the learning of both discriminations. In the test phase, within the framework of the experimental conditions, corticosterone and the vehicle solutions were injected 15 min before the test session.

### Histology

Immediately after behavioral testing (Experiments 1 and 3) or microdialysis measurements (Experiment 2), mice were killed by cervical elongation and decapitated to remove brain which was subsequently soaked in a 10% formaldehyde solution over 10 days. At the end of this period, the brains were soaked in a saccharose-formaldehyde solution (10% formaldehyde solution +30% sucrose) during 2 days. Brains were then sectioned coronally (50 μm thickness in successive slices). A thionin (Sigma, France) stain was used to determine location of the guide-cannulae.

### Statistical analysis

Statistical analyses were performed using the Statview 5.0 software. The data were analyzed using one way (factorial analyses) or Two-Ways (between factors interaction) analyses of variance (ANOVAs) followed, whenever adequate, by *post-hoc* comparisons (Bonferroni/Dunnett's test). Data were expressed as means ± s.e.m. Comparisons of retrieval performances with chance level were calculated with one sample Student-t-test (with hypothesized mean = chance level of 50%). Microdialysis data were analyzed using One - or Two-Way repeated-measure ANOVA as appropriate, followed when adequate by *post-hoc* testing (Bonferroni/Dunnett's test). Within group comparisons of values with baseline level was calculated using one sample Student-t-test. “NS” means that “p” values are superior to 0.05 and are considered as non-statistically significant.

## Results

### Experiment 1: effects of intra-dHPC or intra-mPFC lidocaine injections on MRPs in non-stress and stress conditions

#### Impact of lidocaine injections into the dHPC 30 min before memory testing in non-stress and stress conditions

***Acquisition phase***. All animals received the vehicle infusion 30 min before the acquisition phase. The acquisition phases of groups tested for discrimination 1 or 2 have been analyzed according to the latter random attribution of mice to groups (lidocaine or vehicle), conditions (stress vs. non-stress) and D1 or D2 memory testing. No difference between groups was observed on the total number of head-dips (NS in all comparisons).

***Test phase***. The total number of explorations is precised in Table 1. No difference was observed on the total number of head-dips among the groups injected into the dHPC for both discriminations 1 and 2 (see Table 1A).

**Table 1A:** **Total number of explorations during the test phase in dHPC-injected groups attributed to memory testing of the first discrimination and second discriminations**.

**dHPC**	**Discrimination 1**	**Discrimination 2**
**A Groups**	**Total number of head-dips mean ± s.e.m**.	**Total number of visits mean ± s.e.m**.
Vehide non-stress	15.4 ± 1.1	19.0 ± 2.2
Vehide stress	16.6 ± 1.2	21.9 ± 1.3
Lidocaine non-stress	14.5 ± 0.8	17.1 ± 2.6
Lidocaine stress	15.3 ± 1.6	16.3 ± 1.9

No significant difference between groups was observed.

**Figure 2:**
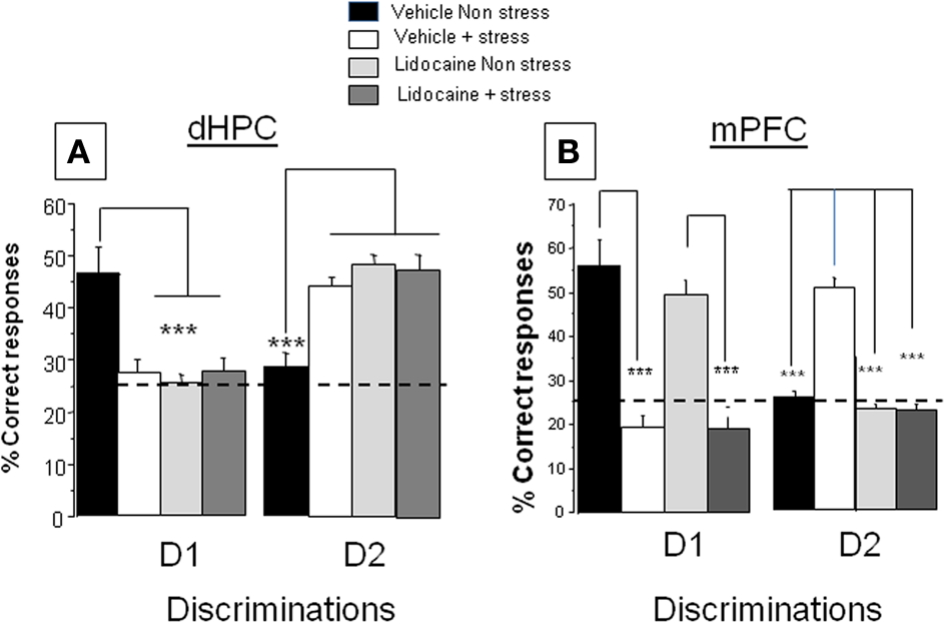
**Effects of lidocaine injections into the dHPC and mPFC on memory patterns in the CSD task in non-stress and stress conditions. (A)** dHPC. *Discrimination 1, D1 Left*: Percentage of correct responses in vehicle and lidocaine-injected mice in non-stress and stress conditions. Lidocaine was injected 15 min before stress delivery (which occurred 15 min before test) thus 30 min before behavioral testing. Stress and lidocaine induced a significant decrease of the % of correct responses (^***^p < 0.001 vs. Vehicle non-stress). **(A)** *Discrimination 2, D2 right*: Percentage of correct responses in vehicle and lidocaine-injected mice in non-stress and stress conditions. Stress and lidocaine induced a significant increase of the % of correct responses (^***^p < 0.001 vs. Vehicle non-stress); **(B)** mPFC: *Discrimination 1, D1: Left*: Percentage of correct responses in vehicle and lidocaine-injected mice in non-stress and stress conditions. Stress significantly decreased the % of correct responses (^***^p < 0.001 vs. Vehicle non-stress); in contrast, lidocaine had no effect in non-stress condition as compared the Vehicle non-stress group (NS) and lidocaine did not blocked the deleterious effect of stress on performance (Lidocaine+stress vs. Lidocaine non-stress; ^***^p < 0.001). *Discrimination 2, D2: right:* Percentage of correct responses in vehicle and lidocaine-injected mice in non-stress and stress conditions. Stress induced a significant increase of the % of correct responses (^***^p < 0.001 vs. Vehicle non-stress). Lidocaine blocked the stress-induced increase of the % of correct responses (^***^p < 0.001 vs. Vehicle+stress).

*Percentage of correct responses (A) first discrimination (D1)*. Data are represented in Figure 2A, left. ANOVA evidenced a significant difference between groups [*F*_(3, 30)_ = 6.5; *p* < 0.001]. More specifically, in non-stress condition, lidocaine injection induced an impairment of D1 responses (25.2 ± 2.0%) as compared to vehicle–injected mice (46.4 ± 5.0%; *p* < 0.001); stress induced a decrease of D1 responses (27.2 ± 2.6%); as compared to non-stressed animals (46.4 ± 5.0%; *p* < 0.001). Lidocaine injected before stress delivery did not modify performance as compared to non-stressed lidocaine injected mice (27.5 ± 2.9% and 25.2 ± 2.0% respectively; NS) or as compared to stressed vehicles (27.2 ± 2.6%; NS).

*Percentage of correct responses. (B) second discrimination (D2)*. Data are represented in Figure 2A, right. ANOVA showed a significant difference between groups [*F*_(3, 30)_ = 12.3; *p* < 0.0001]. More specifically, in non-stress condition, lidocaine injection before test induces an increase of D2 (49.7 ± 2.4%) as compared to mice receiving the vehicle solution (28.9 ± 2.2%; *p* < 0.001); stress induced an increase of D2 (44.5 ± 2.0%) as compared to non-stressed animals (28.9 ± 2.2%; *p* < 0.001). Lidocaine injected before stress delivery (48.3 ± 3.2%) did not modify performance as compared to lidocaine-injected mice (49.7 ± 2.4%; NS) or stressed vehicles (44.5 ± 2.0%; NS).

#### Impact of lidocaine injections into the mPFC 30 min before memory testing in non-stress and stress conditions

***Acquisition phase***. All animals received the vehicle infusion 30 min before the acquisition phase. The acquisition phase has been analyzed according to the latter random attribution of mice to groups, conditions (stress vs. non-stress) and D1 or D2 retrieval test phases. No difference was observed on exploratory patterns among groups (NS in all comparisons).

***Test phase***. The total number of explorations is precised in Table 1. No difference was observed on the total number of head-dips among the groups injected into the mPFC for both discriminations 1 and 2 (see Table 1B).

**Table 1B:** **Total number of explorations during the test phase in mPFC-injected groups attributed to memory testing of the first discrimination and second discriminations**.

**mPFC**	**Discrimination 1**	**Discrimination 2**
**B Groups**	**Total number of head-dips mean ± s.e.m**.	**Total number of visits mean ± s.e.m**.
Vehide non-stress	17.1 ± 3.3	16.6 ± 4.2
Vehide stress	19.7 ± 2.8	20.2 ± 4.3
Lidocaine non-stress	20.1 ± 1.1	18.7 ± 3.9
Lidocaine dress	18.6 ± 3.6	17.1 ± 4.1

No significant difference between groups was observed.

*Percentage of correct responses. (A) first discrimination (D1)*. Data are represented in Figure 2B, left. ANOVA showed a significant difference between groups [*F*_(3, 32)_ = 8.67; *p* < 0.0001]. More specifically, stress induced a significant decrease of D1 responses (Stressed vehicles: 19.4 ± 4.6%) as compared to non-stressed vehicles (55.5 ± 5.5%; *p* < 0.001). Lidocaine injection before stress did not modify performance (20.1 ± 2.4%) as compared to stressed vehicles (19.4 ± 4.6%; NS) whereas non-stressed lidocaine injected mice (49.2 ± 2.9%) behaved similarly to non-stressed vehicles (55.5 ± 5.5%; NS).

*Percentage of correct responses. (B) second discrimination (D2)*. Data are represented in Figure 2B, right. ANOVA showed a significant difference between groups [*F*_(3, 32)_ = 7.9; *p* < 0.0001]. More specifically, stress induced a significant increase in the % number of D2 responses (stressed vehicles: 51.3 ± 2.2%) as compared to non-stressed vehicles (26.2.0 ± 2.1%; *p* < 0.001). Lidocaine injection before stress significantly decreased the % number of D2 responses (24.5 ± 1.6%) as compared to stressed vehicles (*p* < 0.001) whereas non-stressed lidocaine-injected mice (23.9 ± 1.8%) behaved similarly to non-stressed vehicles (26.2 ± 2.1%; NS).

*Conclusion*. This experiment shows (i) that the MRP in stress conditions (D2 better retrieved than D1) is inversed as compared to the non-stress conditions (better D1 retrieval than D2); (ii) that the memory retrieval of D1 under non-stress conditions is sustained by the dHPC activity, whereas the emergence of D2 within stress conditions involves the mPFC activity.

### Experiment 2: plasma corticosterone and time-course evolutions of corticosterone concentrations after stress into the dHPC and mPFC

#### Plasma corticosterone assay

**Figure 3:**
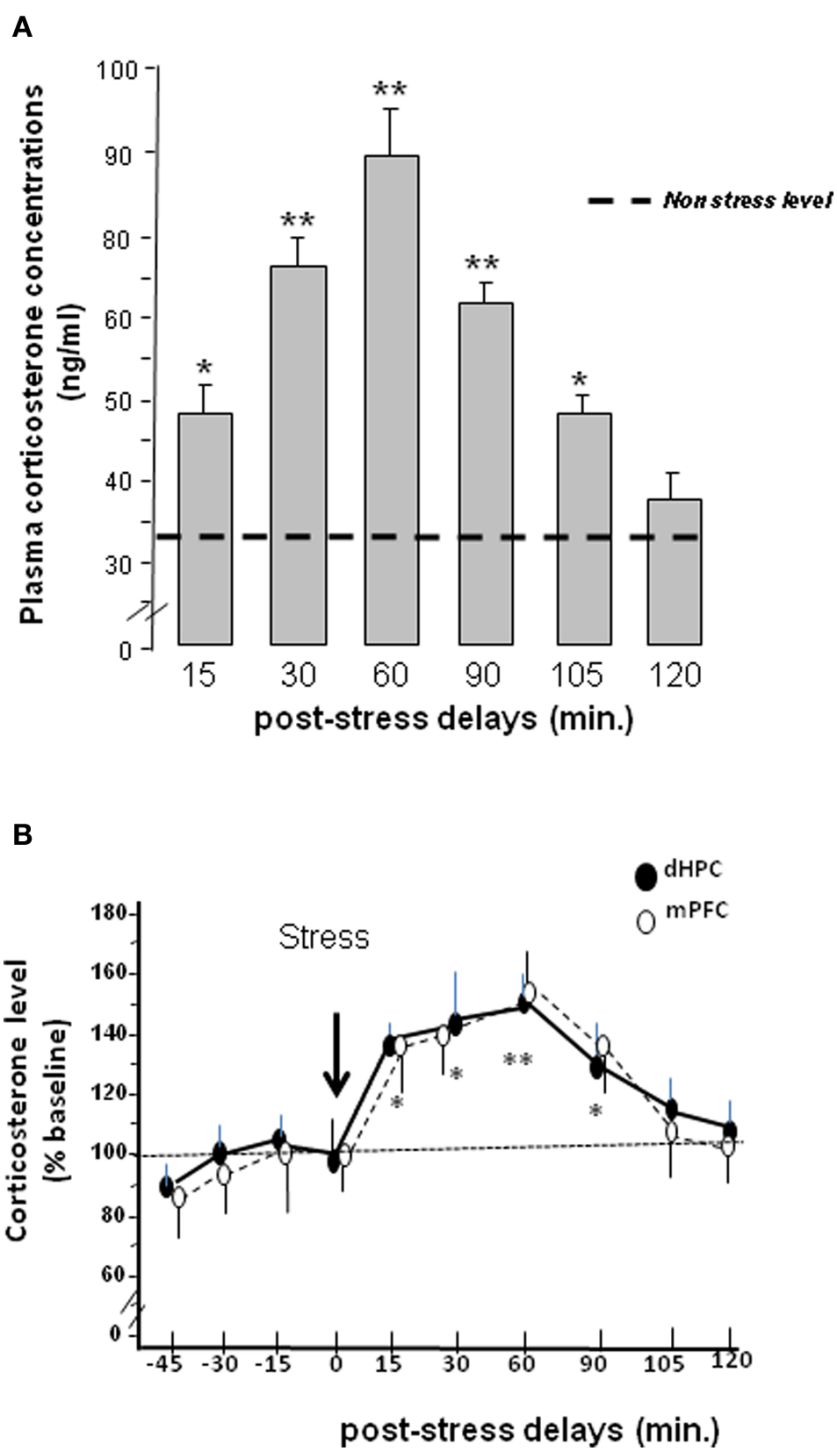
**(A)** Plasma concentrations (expressed in ng/mL) at different post-tress delays after the occurrence of the acute stress. (^*^p < 0.05 and ^**^p < 0.01 vs. non-stress level). **(B)** Intracerebral double-microdialysis. Time-course evolutions of stress-induced corticosterone rises within the dHPC (dark circles) and mPFC (white circles) measured by microdialysis in the same animal. In dHPC and mPFC, the time-course evolutions of corticosterone are similar. In both brain areas, corticosterone levels significantly differed from baseline from the 15 min post-stress delay to the 90 min one. The maximum corticosterone concentration level is measured at the 60 min post-stress delay followed by a return to baseline 105 min after stress delivery. Results are expressed in relative concentrations. No significant difference between groups was observed (NS). Comparisons to baseline for dHPC and mPFC: ^*^p < 0.05; ^**^p < 0.01.

Data are represented in Figure 3A. ANOVA showed a significant difference between groups [*F*_(6, 42)_ = 8.75; *p* < 0.001]. More specifically, stress induced a significant increase of plasma corticosterone level at the post-stress delays of 15 min (49.8 ± 3.1 ng/ml; *p* < 0.05), 30 min (67.2 ± 5.4 ng/ml; *p* < 0.01), 60 min (89.1 ± 11.3 ng/ml; *p* < 0.01), 90 min (62.8 ± 4.9 ng/ml; *p* < 0.01) and at 105 min (47.1 ± 1.48 ng/ml; *p* < 0.05) as compared to non-stressed animals (34.2 ± 3.1 ng/ml). By contrast, the increase in corticosterone at the 120 min post-stress delay (38.7 ± 5.3 ng/ml) was not significantly different from that observed in non-stressed mice (*p* > 0.10).

#### Time-course evolution of corticosterone concentrations after stress into the dHPC or mPFC

**Figure 4:**
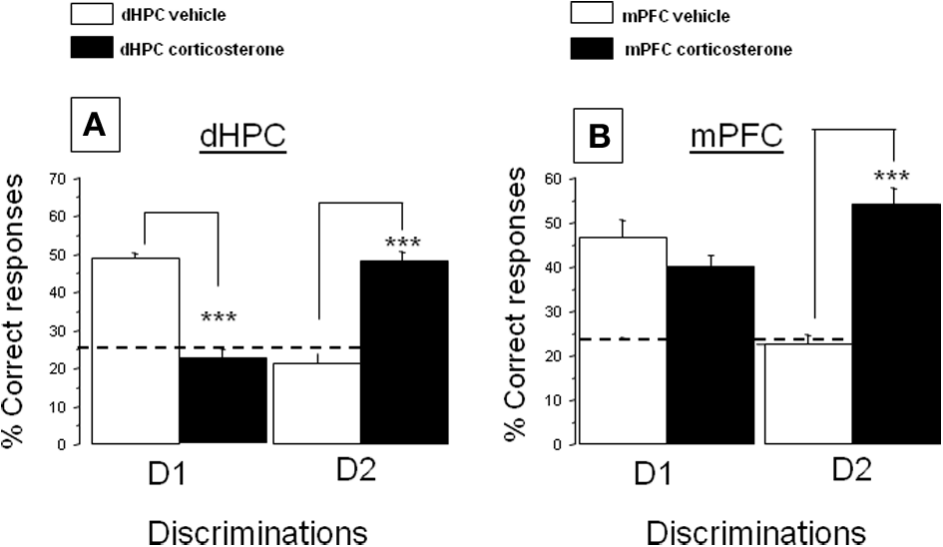
**Effect of corticosterone injections into the dHPC or mPFC on the % correct responses in the CSD task**. **(A)** dHPC First discrimination, D1: corticosterone injected into the dHPC significantly decreases the % correct responses as compared to vehicle-injected mice (^***^p < 0.001); Second discrimination, D2: corticosterone injected in the dHPC increased the % of correct responses as compared to vehicle-treated control group (^***^p < 0.001); **(B)** First discrimination, D1: corticosterone injected into the mPFC has no significant effect as compared to vehicle-treated group; Second discrimination, D2: corticosterone injected into the mPFC increased the % of correct responses as compared to the vehicle-treated control group (^***^p < 0.001).

Data are represented in Figure 3B. The absolute concentrations of baseline corticosterone levels in dialysates (i.e., mean ± s.e.m. from 4 points measured before stress delivery) are not significantly different between dHPC and mPFC groups [386.9 ± 39.5 ng/l vs. 332.5 ± 39.8 ng/l respectively; *F*_(1,12)_ < 1.0; NS]. Figure 4B represents corticosterone levels in dHPC and mPFC areas in the same animals (*N* = 7) in which data are expressed in relative concentrations (i.e., as percentage of variation of baseline).

Repeated-measure ANOVAs performed on corticosterone kinetic evidenced a significant evolution of corticosterone over delays [*F*_(9, 108)_ = 5.3; *p* < 0.0001] but a non-significant interaction between brain areas and delays [*F*_(9, 108)_ = 0.37; *NS*]. Thus, the time-course evolutions of corticosterone rises in dHPC and mPFC groups after acute stress delivery are very similar. More specifically, stress induced in dHPC a progressive and significant increase in corticosterone levels as compared to the last pre-stress sample (92.5 ± 6.2%; “time = 0”), from 15 min (136.7 ± 5.2%; *t* = 2.38; *p* < 0.05) to 90 min (121.1 ± 4.1%; *t* = 2.58; *p* < 0.05). Furthermore, the highest difference was observed 60 min after stress administration (146.1 ± 4.7%; *t* = 3.57; *p* < 0.01). In the mPFC, stress induced a progressive and significant increase in corticosterone levels as compared to the last pre-stress sample (98.8 ± 3.9%; “time = 0”), from 15 min (135.2.4 ± 8.6%; *t* = 2.48; *p* < 0.05) to 90 min (135.1 ± 9.2%; *t* = 2.82; *p* < 0.05). Furthermore, the highest difference was also observed 60 min after stress delivery (154.5 ± 7.4%; *t* = 3.49; *p* < 0.01).

***Conclusion***. Stress induced significant time-course evolutions of corticosterone rises in both the mPFC and dHPC, which are similar in both brain structures.

### Experiment 3: effects of dHPC or mPFC corticosterone injections on MRPs in the CSD task

Given the data obtained in Experiments 1 and 2, we hypothesized that corticosterone likely enhances the mPFC-dependent MRP and impaired the dHPC-dependent one. To appraise such a hypothesis, we injected corticosterone within the dHPC or the mPFC 15 min before memory testing. An ANOVA showed a significant difference between group on the % of correct responses both for the first [*F*_(3, 33)_ = 19.4; *p* < 0.0001] and second discrimination [*F*_(3, 32)_ = 37.02; *p* < 0.0001].

#### Impact of corticosterone injection into the dHPC on the memory retrieval of the first and second discriminations

***Acquisition phase***. All animals received the vehicle infusion 15 min before the acquisition phase. The acquisition phases of groups tested for discrimination 1 or 2 have been analyzed according to the latter random attribution of mice to groups (corticosterone or vehicle) and D1 or D2 retrieval test phases. No difference was observed on account of the total number of head-dips among the groups (NS in all comparisons).

***Test phase***. The total number of explorations is mentioned in Table 2. No difference was observed on the total number of head-dips among the groups injected into the dHPC for both discriminations 1 and 2 (see Table 2A).

*(A) First discrimination (D1).* Data are displayed in Figure 4A, left. More precisely, intra-dHPC injections of corticosterone induced a significant decrease of D1 responses (22.2 ± 3.0%) as compared to respective dHPC vehicles (48.9 ± 1.3%; *p* < 0.001).
*(B) Second discrimination (D2).* Data are represented in Figure 4A, right. More specifically, intra-dHPC injections of corticosterone induced a significant increase of D2 responses (48.5 ± 2.5%) as compared to dHPC vehicles (21.4 ± 2.6%; *p* < 0.001).

**Table 2A:** **Total number of explorations during the test phase in dHPC-injected groups attributed to memory testing of the first discrimination and second discriminations**.

**A dHPC**	**Discrimination 1**	**Discrimination 2**
**Groups**	**Total number of head-dips mean ± s.e.m**.	**Total number of head-dips mean ± s.e.m**.
Vehicle	17.5 ± 1.9	20.4.± 3.6
Corticosterone	19.3 ± 2.2	18.9 ± 4.3

No significant difference between groups was observed.

#### Impact of corticosterone injection into the mPFC over memory retrieval of the first and second discriminations

***Acquisition phase***. All animals received the vehicle infusion 15 min before the acquisition phase. The acquisition phases of groups tested for discrimination 1 or 2 have been analyzed according to the latter random allocation of mice to groups (corticosterone or vehicle) and D1 or D2 retrieval test phases. No difference was observed on the total number of head-dips among the groups (NS in all comparisons).

***Test phase***. The total number of explorations is mentioned in Table 2B. No difference was observed on the total number of head-dips among the groups injected into the mPFC for both discriminations 1 and 2.

**Table 2B:** **Total number of explorations during the test phase in mPFC-injected groups attributed to memory testing of the first discrimination and second discriminations**.

**B mPFC**	**Discrimination 1**	**Discrimination 2**
**Groups**	**Total number of head-dips mean ± s.e.m**.	**Total number of head-dips mean ± s.e.m**.
Vehicle	19.4 ± 3.2	23.4 ± 3.2
Corticosterone	22.6 ± 4.1	20.5 ± 2.8

No significant difference between groups was observed.

*First discrimination*. Data are displayed in Figure 4B, left. Corticosterone did not decrease the % of D1 correct responses as compared to controls (47.8 ± 3.2% vs. 54.4 ± 4.4%, respectively; NS).

*Second discrimination*. Data are displayed in Figure 4B, right. Intra-mPFC injections of corticosterone induced a significant increase of D2 responses (54.1 ± 3.5%) as compared to controls (22.4 ± 2.2%; *p* < 0.001).

### Histology

**Plate 1:**
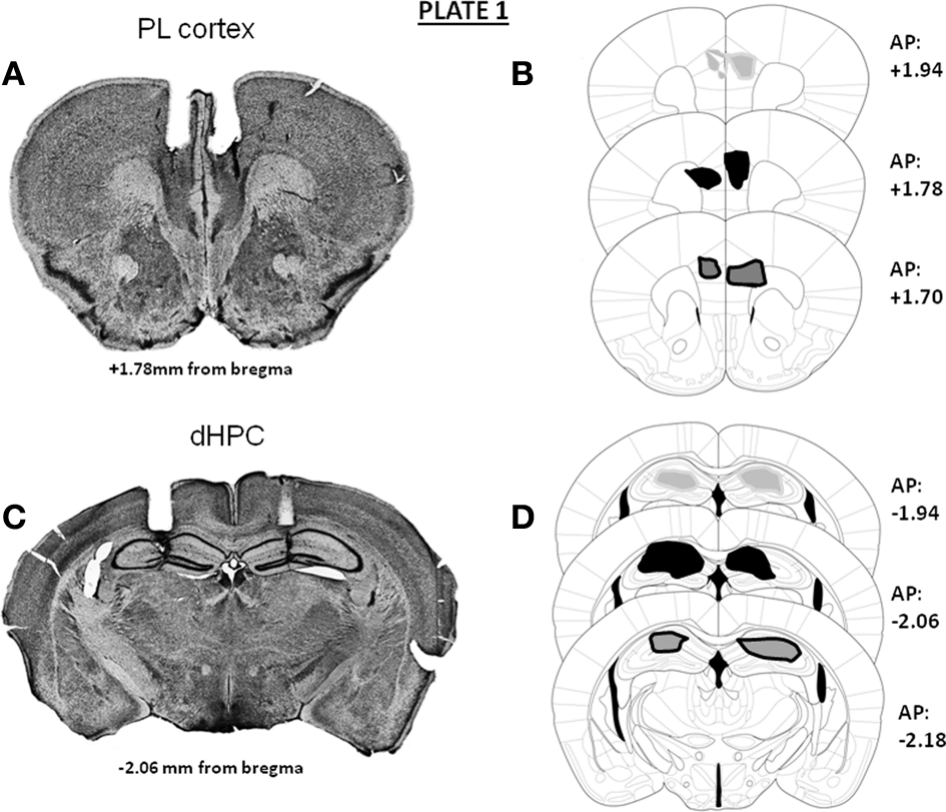
**Representative photomicrographs and antero-posterior extent of the double bilateral localizations of cannulae for pharmacological injections in Experiments 1 and 3 in the prelimbic cortex (A,B) or in the dorsal hippocampus (C,D); the densities of cannulae localizations are represented in light gray area (20%), dark area (50%) and dark gray area (30%) On the right of each diagram, stereotaxic anterio-posterior (AP) coordinates are mentioned (in mm from bregma)**.

**Plate 2:**
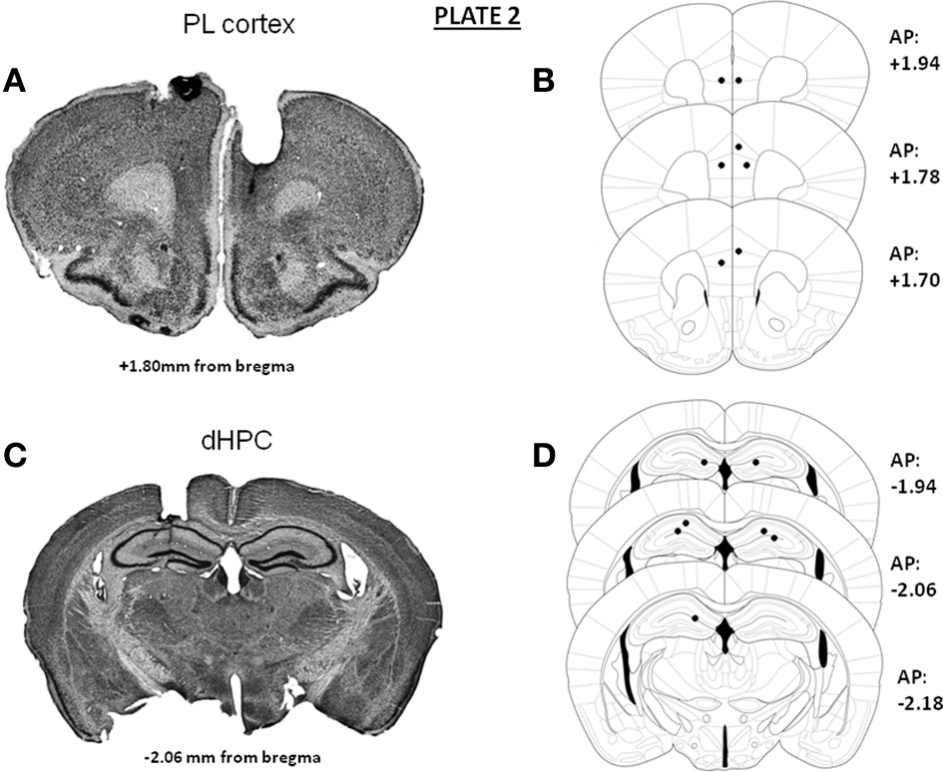
**Representative photomicrographs and antero-posterior extent of the unilateral implantations of cannulae into the prelimbic cortex (A,B) and the dorsal hippocampus (C,D) in the same animal for the microdialysis study**. On the right of each diagram, stereotaxic anterio-posterior (AP) coordinates are mentioned (in mm from bregma).

In Experiments 1 and 3, independent groups of animals were implanted bilaterally either in the mPFC (prelimbic cortex) or in the dHPC. Representative microphotographs and the antero-posterior extent of the bilateral canulae localizations into either the mPFC (A) or dHPC implantations (B) in animals used in Experiments 1 and 3 are provided in Plate 1. In Experiment 2 (microdialysis), guide-cannulae were implanted unilaterally in the same animal, one canulae into the mPFC and the other into the dHPC, at the same stereotaxic coordinates as those used in Experiments 1 and 3. Implantations into the mPFC and the dHPC were alternated from one mouse to the other, to avoid possible laterality biases. Representative microphotographs and the antero-posterior extent of the canulae localizations into the mPFC (A and C) and dHPC (B and D) in animals used for the microdialysis experiment are provided in Plate 2.

## Discussion

To sum up our findings, we showed in a first experiment that stress (electric footshocks) inversed the MRP in the CSD task as compared to non-stress conditions. Lidocaine injections 30 min before memory testing into either the mPFC or dHPC revealed that response patterning in non-stress conditions is sustained by dHPC activity whereas under stress conditions, it depended on mPFC activity. Thus, stress enhanced the mPFC-dependent MRP at the expense of the dHPC-dependent one. It is noteworthy indeed that *in vivo* double-microdialysis, provided unequivocal evidence to the effect that acute stress induced very similar time-course evolutions of corticosterone rises into the mPFC and dHPC. Thus, the stress-induced corticosterone rises at the time of memory testing in both areas were concomitant with the emergence of the mPFC-dependent MRP and the blockade of the dHPC one. In keeping with these findings, we tested in Experiment 3 the hypothesis that the corticosterone rise in the mPFC and dHPC may account for the blockade of the dHPC-dependent MRP and the concomitant emergence of the mPFC one after stress. Indeed, we found that *in situ* injections of corticosterone into either the mPFC or the dHPC both enhanced the mPFC-dependent MRP and impaired the dHPC-dependent one.

### Neural networks in the CSD task under non-stress and stress conditions

In the present study, the first experiment confirmed our previous findings showing that stress modifies the serial MRPs as compared to non-stressed mice (Chauveau et al., 2008, 2009; Tronche et al., 2010). More specifically, whereas memory retrieval of the first learned discrimination is predominant as compared to the second one in non-stress conditions, stress reversed this MRP, the second discrimination being better retrieved than the first one. Using selective inactivation of the mPFC or dHPC with lidocaine, we showed that the retrieval of information in stress condition recruited the mPFC whereas the dHPC is engaged in non-stress conditions. Indeed, in non-stress conditions, the retrieval of D1 is not impaired by lidocaine injection into the mPFC as compared to vehicle-treated mice; in contrast, the retrieval of D1 is severely impaired by lidocaine injection into the dHPC. Thus, in non-stress conditions, memory retrieval of D1 is critically dependent on dHPC but not on mPFC activity. The opposite is observed in stress conditions for D2 responses. Indeed, stress increases the memory retrieval of D2 at the expense of D1; however, whereas lidocaine injection into the dHPC did not impair memory retrieval of D2, it totally blocked the emergence of D2 in stressed animals when injected into the mPFC. These overall data led us to conclude that the emergence of D2 responses in stressed mice was dependent on the mPFC but not on dHPC activity. This conclusion is further sustained by the fact that lidocaine injected into the mPFC did not modify D1 responses both in stress and non-stress conditions as compared to vehicles. As further evidence, lidocaine injections into the dHPC did not modify D2 responses both in stress and non-stress conditions, as compared to vehicles. Interestingly, we found that the different effects of lidocaine were transient, since they were observed when injected 30 min before memory testing as reported here, but not when injected 120 min before behavioral testing (*data not shown*).

Overall, our data released in our present study, prove singularly congruent with several studies showing that hippocampal lesions resulted in an initial retention deficit of an object-spatial location association (the equivalent of D1 in our study) and spared performance for a more recent one (the equivalent of D2 in the present study) (Jackson et al., 1998; Gilbert and Kesner, 2004). Our present data are also in agreement with studies showing that the dHPC and the mPFC play significant roles in memory for the serial order of spatial and non-spatial information (Fortin et al., 2002; Kesner et al., 2002; Hannesson et al., 2004; Lisman et al., 2005; Kesner and Hopkins, 2006). Interestingly, several studies have shown that there are multiple ways by which the dHPC and mPFC interact during memory testing; thus, a time-dependent sequential involvement of the HPC and mPFC in consolidation processes has been already evidenced (Frankland and Bontempi, 2005); however, our present study provides further evidence that the mPFC and the dHPC can also compete at the time of memory testing and that the expression of the dHPC or mPFC-dependent response depends on the conditions (non-stress vs. stress) in which memory retrieval occurred.

### Stress, corticosterone, and memory retrieval

The role of corticosterone in the stress-induced inversion of MRP was already demonstrated in a previous study, in which we reported that the injection of metyrapone (an inhibitor of corticosterone synthesis) before stress delivery totally blocked the stress-induced inversion of the MRP in the CSD task (Chauveau et al., 2010). Our present study provides clear-cut evidence that the simultaneous stress-induced increase of corticosterone in the mPFC and dHPC generates opposite effects on mPFC and dHPC-dependent MRP. Indeed, we evidenced simultaneous corticosterone rises into both the dHPC and mPFC after stress delivery associated with both the emergence of the mPFC–dependent response and the parallel alteration of the dHPC-dependent one.

Interestingly, corticosterone injections did not exactly mimic the effect of stress on memory retrieval. Indeed, when corticosterone is injected into the mPFC, both D1 and D2 are well remembered; in contrast, stress impaired D1 and enhanced D2. This discrepancy may be explained by the fact that stress increases corticosterone concentrations both in the mPFC and the dHPC which resulted in a simultaneous enhancement of D2 and an impairment of D1. In contrast, the corticosterone injection specifically into the mPFC spared D1 response (sustained by dHPC activity) but enhanced D2 one.

The stress-induced rise of corticosterone into the dHPC plays a key role in the alteration of the dHPC-dependent response. Indeed, from a cognitive point of view, the effect of corticosterone rise into the dHPC is similar to that induced by the injection of lidocaine (Experiment 1) which resulted in a decrease of D1 response and an enhancement of the D2 one. Thus, an increased level of corticosterone into the dHPC results in an alteration of memory retrieval sustained by dHPC activity. This result is congruent with studies showing that the memory retrieval impairment induced by hippocampal CA3 lesions is blocked by adrenocortical suppression,—a finding which suggest that elevated adrenocortical activity is critical in mediating the memory deficits induced by HPC damage (Roozendaal et al., 2001). In contrast, the stress-induced corticosterone rise in the mPFC or direct injection of corticosterone into the mPFC in non-stressed animals induced an enhancement of D2 responses, i.e., favored the emergence of the mPFC-dependent MRP. This finding is in sharp contrast with studies having reported that GCs can disrupt mPFC–dependent memory (Roozendaal et al., 2004b; Cerqueira et al., 2005). However, the effect of corticosterone on memory depends on interactions with other local neurotransmitters within the mPFC; indeed, it has been already reported that endogenous GCs are essential for maintaining prefrontal cortical cognitive function by interacting with the dopaminergic D_1_ receptor (Mizoguchi et al., 2004); more recently, GCs in the prefrontal cortex of rats have been reported to enhance memory consolidation whereas they impaired working memory by a common neural mechanisms, which critically depended on the interaction with the noradrenergic activity within the prefrontal cortex (Barsegyan et al., 2010). Another study reported an improvement of working memory after stress due to an enhancement of glutamatergic transmission in the prefrontal cortex (Yuen et al., 2009). Thus, the opposite effect of corticosterone on mPFC and dHPC functions could be mediated either by the interaction of corticosterone with other local neurotransmitters or by regional differences in MR and GR receptors densities within each area. It is indeed well established that corticosterone alters neural plasticity via an increase of phospho-CREB levels which depend on the activation of membrane-associated glucocorticoid receptors (Roozendaal et al., 2010). Thus, low (non-stress condition) and high (stress condition) levels of corticosterone concentrations definitely affect regional neural plasticity as well as the interaction between GCs and dopaminergic, glutamatergic, and GABAergic transmissions, which normally equilibrate excitation and inhibition within the HPC-PFC network (see in Curley and Lewis, 2012; Godsil et al., 2013). Further studies will be performed to ascertain the impact of the stress-induced corticosterone rises on brain regional neural plasticity using immunohistochemical approaches.

In conclusion, by using a dynamic approach of the time-course evolution of corticosterone levels our study herein evidenced that acute stress induced similar corticosterone rises in the mPFC and dHPC over time, that produced a shift from dHPC-dependent MRP (non-stress condition) to mPFC–dependent one. By and large, our study demonstrates that corticosterone bears differentiated functional effects on the activity of the dHPC and mPFC, and is essential in mediating the deleterious effects of stress on the mPFC-HPC interplay.

## Disclosure

We wish to extend the following statements: except for income received from primary employers, no financial support nor any compensation has been received from either any individual or corporate entity over the past 3 years whether dedicated to either research or professional service in relation with this study. Further, no single personal financial holding may exist nor be perceived as constituting a potential conflict of interest. All authors of the study display no conflict of interest.

### Conflict of interest statement

The authors declare that the research was conducted in the absence of any commercial or financial relationships that could be construed as a potential conflict of interest.
